# Cellular distribution of C-C motif chemokine ligand 2 like immunoreactivities in frontal cortex and corpus callosum of normal and lipopolysaccharide treated animal

**DOI:** 10.1186/s12868-022-00706-y

**Published:** 2022-03-30

**Authors:** Xue Shi, Xinrui Gong, Huangui Xiong, Jingdong Zhang

**Affiliations:** 1 Department of Anesthesiology, Tongji Hospital, Tongji Medical College, Huazhong University of Science and Technology, Wuhan, 430030 China; 2 Department of Anesthesiology, Xiangyang Central Hospital, Affiliated to Hubei University of Arts and Science, 136 Jinzhou Street, Xiangyang, 441021 China; 3 Department of Pharmacology and Experiment Neuroscience, University of Nebraska Medical Center, Omaha, NE 68198-5880 USA; 4 Department of Anesthesiology, University of Cincinnati College of Medicine, Cincinnati, OH 45267-0531 USA

**Keywords:** CCL2 like immunoreactivity, CCL2 cellular distribution, Cortex grey matter, Corpus callosum white matter, Perivascular areas

## Abstract

**Background:**

C-C motif chemokine ligand 2 (CCL2) is reported to be involved in the pathogenesis of various neurological and/or psychiatric diseases. Tissue or cellular expression of CCL2, in normal or pathological condition, may play an essential role in recruiting monocytes or macrophages into targeted organs, and be involved in a certain pathogenic mechanism. However, few studies focused on tissue and cellular distribution of the CCL2 peptide in brain grey and white matters (GM, WM), and the changes of the GM and WM cellular CCL2 level in septic or endotoxic encephalopathy was not explored. Hence, the CCL2 cellular distribution in the front brain cortex and the corpus callosum (CC) was investigated in the present work by using immunofluorescent staining.

**Results:**

(1) CCL2 like immunoreactivity (CCL2-ir) in the CC is evidently higher than the cortex. When the measurement includes ependymal layer attached to the CC, CCL2-ir intensity is significantly higher than cortex. (2) Structures in perivascular areas, most of them are GFAP positive, contribute major CCL2-ir positive profiles in both GM and WM, but apparently more in the CC, where they are bilaterally distributed in the lateral CC between the cingulate cortex and ventricles. (3) The neuron-like CCL2-ir positive cells in cortex are significantly more than in the CC, and that number is significantly increased in the cortex following systemic lipopolysaccharide (LPS), but not in the CC. (4) In addition to CCL2-ir positive perivascular rings, more CCL2-ir filled cashew shape elements are observed, probably inside of microvasculature, especially in the CC following systemic LPS. (5) Few macrophage/microglia marker-Iba-1 and CCL2-ir co-labeled structures especially the soma is found in normal cortex and CC; the co-localizations are significantly augmented following systemic LPS, and co-labeled amoeba like somata are presented. (6) CCL2-ir and astrocyte marker GFAP or Iba-1 double labeled structures are also observed within the ependymal layer. No accumulation of neutrophils was detected.

**Conclusion:**

There exist differences in the cellular distribution of the CCL2 peptide in frontal cortex GM and subcortical WM–CC, in both the physiological condition and experimental endotoxemia. Which might cause different pathological change in the GM and WM.

**Supplementary Information:**

The online version contains supplementary material available at 10.1186/s12868-022-00706-y.

## Background

Chemokine C-C motif ligand 2 (CCL2), also termed monocyte chemoattractant protein 1, is known as a pivotal chemoattractant for immune-cells, especially monocytes and T-cells, and is involved in pathogenesis of a number of diseases [1–4]. Some previous studies have shown that the CCL2 synthesized in tissues or cells induced by inflammatory stimulations played an essential role in recruiting monocytes or macrophages into the targeted organs or tissues [2–4]. In murine model, intervertebral disc herniation upregulated the CCL2 expression and induced inflammatory pain [2]. Aortic vascular smooth muscle of the hypertensive rats expressed significant higher level of the CCL2 [3], which recruits lipid-filled foam macrophages to accumulate in situ and to cause atherosclerosis [4]. On the other hand, CCL2 receptor CCR2 knocked out could protect renal injury in murine renovascular hypertension, and ameliorate the white matter injury during experimental autoimmune encephalomyelitis—an animal model for studying multiple sclerosis [5, 6]. A recent human study showed that one of pathogenic factors for coronavirus associated cardiac injury is related to CCL2 over-expression in myocardial and/or interstitial cells evoked by virus. The over-expressed CCL2 recruits excessive macrophages that engulf the host cells, and the healthy cardiac cells as well [7]. Therefore, illustrating the cellular expression of CCL2 is significant for understanding the mechanism of pathological changes in certain disease, and may benefit for developing tissue or cell targeted therapeutic approaches against the pathogenic CCL2 [8, 9].

However, the data about CCL2 tissue distribution in the brain and the relationship of this distributive pattern with observed pathological change are still sparse. A set of previous studies indicated that the CCL2 level is higher in the brain white matter (WM) than in the grey matter (GM) [10–12]. For instance, a study in healthy C57BL/6 J mice displayed the basic CCL2 mRNA expression in the corpus callosum (CC) WM was about 300-times higher compared to that found in the neighboring cortex [10]. Utilizing Western blot and ELISA, we have shown that the concentration of CCL2 peptide was significantly higher in the brain WM than in the neighboring GM in naïve rats and neurologically health humans [11]. In cases of multiple sclerosis, robust T-cells and macrophages infiltration were observed in the WM, but less frequently in the GM [12, 13]. We also observed in simian immunodeficiency virus (SIV) infected brain that the number of perivascular macrophage cuffing (PC) in the WM was significantly higher than in the GM [14]. Significantly, an autopsy study on an iatrogenic human immunodeficiency virus infected case who deceased shortly after accidently inoculation showed that the PCs formed fairly early, in a short period after infection [15]. Hence, the higher number of early formed PCs in the WM is possibly related to the higher physiological CCL2 concentration therein [10, 11], rather than the direct effect of the virus [14, 16].

As aforementioned, illustration of in situ cellular distribution of the CCL2 would be beneficial for understanding the mechanism of certain pathological changes. As we have known, the CCL2 plays significant role in sepsis and/or endotoxemia related organs’ injuries [17, 18]; however, there were few studies that compare the cellular CCL2 distribution in the brain GM and the WM of normal subjects and the subjects suffering from sepsis or endotoxemia. Meanwhile, systemic lipopolysaccharide (LPS) is a well-established animal model for the investigation of sepsis and/or endotoxemia related organ-injuries [18–20]. Therefore, in the present work, we attempted to explore cellular CCL2 distribution in the brain GM and the WM of both normal and LPS treated animals. We carried out CCL2 and astrocyte marker double immunostaining at first to detect the peptide levels in the brain of naïve, saline and LPS injected animals, since expression of CCL2 mRNA or peptide have been frequently identified in cultured astrocytes [21, 22]. Meanwhile, we examined CCL2 labeling in microglia/macrophage and/or neuron, and the potential changes following the systemic LPS. In addition, we traced migration states of neutrophils in the GM and the WM, especially around brain vasculature, using a couple of neutrophil markers, considering they equip Toll-like receptors and upgrade expression of CCL2 receptors after LPS binding to the Toll-like receptors [23, 24].

## Materials and methods

### Animals and drugs

Total 14 C57BL/6J mice (8 ~ 10 weeks; 8 male and 6 female), a background strain for many transgenic preparations including CCR2^−/−^ mice [25], were used in this study. Two male and 2 female mice were used as normal control, and to determine the best approach of CCL2 immunofluorescent staining; four mice received intraperitoneal (*i.p.*) injection of sterilized saline (2 males/2 females); and six for LPS injection (2 males used to figure out the time of neuroinflammation occurrence; two males/2 females for collecting analyzable data of immunostaining). All experimental protocols and animal care were carried out in line with the *European Union guideline for Laboratory Animal Care and Use* and approved by the Institutional Research and Ethic Committee of the Tongji medical College, Huazhong University of Science and Technology. All efforts were made to minimize animal suffering and the number of animals used in the study.

The LPS (*Escherichia coli*, L2880, Sigma, St Louise, MO) was prepared with a saline solution at 2 mg/ml, and stored at − 20 °C before using. The animals received a saline (in equal volume to the LPS) or an LPS injection once per day for 5 consecutive days. The LPS was injected at a dose about 1.5 mg/kg. The animal was euthanized one day after the final injection of the saline or LPS.

### Immunofluorescent staining

#### Immunostaining

The animals were euthanized with an overdose of sodium pentobarbital and immediately perfused with saline and followed by a 10% formalin phosphate (Fisher Chemicals) transcardially. The brains were removed, post-fixed overnight, and were cryo-protected by gradient (10% to 30%) sucrose in phosphate-buffered saline (PBS; pH 7.2 ~ 7.4). Coronal frozen sections (14 µm) from the naive, the saline and the LPS injected mice were mounted on the same single Plus^+^ slides (see Additional file 1: Fig. S1A) those were immediately saved in a − 80 °C freezer. Once stained, an extra section in each slide was isolated by Super PAP Pen as a control section (see Additional file 1: Fig. S1A), on which all procedures were the same except the primary antibody was omitted. After routine immune-blocking, sections were incubated in mouse or rabbit anti-CCL2 antibody (see a list of antibodies in Table 1) overnight. For double staining, either mouse or goat anti-glial fibrillary acidic protein (GFAP) antibody were combined with rabbit anti-CCL2 to detect double labeling. Polyclonal goat anti-ionizing calcium binding adaptor protein-1 (Iba-1) was used to reveal the macrophages and the microglia. Polyclonal goat anti-neuron specific nuclear protein (NeuN) was applied to identify the neuron. In addition, either monoclonal rat anti-Ly6g or mouse anti-MPO was used and combined with rabbit anti-CCL2 to explore whether there is a neutrophil accumulation and if any neutrophil is CCL2 positive. Alexa Flour 488 or 594 conjugated anti-primary antibody were applied to visualize the immunofluorescent staining. The sections were mounted with Vectashield mounting medium containing 4',6-diamidino-2-phenylindole (DAPI; Vector Labs).

**Table 1:** Antibodies applied in the current study

Antibodies	Companies	Titers
Monoclonal mouse anti-CCL2, clone 2D8	Sigma (St Louis, MO) MABN712	1:200 ~ 500
Polyclonal rabbit anti-CCL2	Novus (Centennial, CO) NBP1-07035	1:200 ~ 300
Polyclonal rabbit anti-MCP-1	Abcam (Cambridge, MA) AB9899	1:100 ~ 500
Polyclonal rabbit anti-MCP-1	Abcam AB25124	1:100 ~ 500
Polyclonal rabbit anti-MCP-1	Invitrogen (Camarillo, CA) PA5-34505	1:100 ~ 200
Monoclonal mouse anti-GFAP, clone UMAB5	GBI (Mukilteo, WA) UM50005	1:200 ~ 500
Polyclonal goat anti-GFAP	Novus NB 100–53809	1:200 ~ 500
Polyclonal rabbit anti-Iba-1	Wako Chemicals USA Inc. (Richmond, VA)	1:200 ~ 500
Polyclonal goat anti-Iba-1	Abcam AB5076	1:200 ~ 500
Monoclonal mouse anti-NeuN, clone 1B7	Novus NBP1-92693	1:300 ~ 1000
Polyclonal goat anti-NeuN	Novus NBP3-05554–50	1:500 ~ 1000
Monoclonal rat anti-Ly6g, clone RB6-8C5	Santa Cruz (Dallas, TX) SC-53515	1:200 ~ 1000
Monoclonal mouse anti-MPO, clone 266-6K1	Santa Cruz SC-52707	1:100 ~ 1000

MPO: Myeloperoxidase

CCL2 antibody data sheet webpage:

https://www.novusbio.com/products/ccl2-mcp1-antibody_nbp1-07035

https://www.abcam.com/mcp1-antibody-ab9899.html

https://www.abcam.com/mcp1-antibody-ab25124.html

https://www.thermofisher.com/antibody/product/MCP-1-Antibody-Polyclonal/PA5-34505

#### Quantification of CCL2-like immunoreactivity

The CCL2-like immunoreactivity (CCL2-ir) was measured in the same way as we used to quantify the intensity variations of immunofluorescent staining in a previous work [26]. A software SlideBook 6 (Intelligent Imaging Innovations Inc, Denver, CO) was used to measure and analyze the original CCL2-ir intensity and the change in CCL2-ir intensity following systemic LPS, which was then normalized by the whole measured area. The zero level of the CCL2-ir intensity (Additional file 2: Fig. S2A, B and E) was set-up upon control section without primary antibody staining in each slide (Additional file 1: Fig. S1). Initially, we focused on the labeling in the CC WM tract only and the cortex above the CC. Then, the labeling that included the ependymal layer attached to the CC was also selected and measured.

#### Counting double-labeling of CCL2 with GFAP, Iba-1 or NeuN

Afterward, Image J was applied to count double labeled structures through a “multi-point” recording function. Similarly, the number was normalized by the whole measured area of either CC or cortex within the photographed scope field, which is finally converted into the number of cells per mm^2^, based on a 1000-µm scale bar denoted on the original image.

### Verification of CCL2 immunofluorescent staining

Both single Alex Flour 594 (Additional file 2: Fig. S2A, B) and 488 (E) staining were used as control image, and to set up zero-level CCL2-ir intensity, in which the area surrounding the vasculature like profiles, i.e*.* perivascular area (arrows) looked clear, due to without primary antibody. In CCL2 + Iba-1 double labeled section (C and C’), the CCL2-ir labeling can be visualized in perivascular area (C); however, the Iba-1 labeling is clear in perivascular area (C’). Similarly, in CCL2 + NeuN double stained section (D and D’), the higher AF594 intensity was viewed surrounding a blood vessel (aligned arrows in D), but the AF488 is negative in the same area (arrows in D’). In addition, we used AF488 to mark the CCL2 and AF594 to identify the microglia or the neurons, the same pattern of labeling was observed regardless the type of secondary antibody or fluorescent protein (F, F’, G and G’). The CCL2-ir labeling was constantly presented in perivascular area (arrows in F and G) whenever anti-CCL2 is added, and the other primary or secondary antibody or fluorescent proteins did not precipitate in the perivascular area (C’, F’ and G’, D’ aligned arrows).

### Imaging acquisition and processing

Digital images of fluorescent labeling were collected with Keyence BZ-X800E microscope (Keyence Corp. America, Itasca, IL USA) equipped with the SlideBook plug-in. The CCL2-ir intensity data were analyzed through a Dell computer installed with SlideBook 6 processer. And the double labeled cells were observed and captured under 20× objective lens, two scale bars of 100 µm and 1000 µm were denoted on the images, the former is used as scale bar in publishable figures and the latter is for convert area size into the square millimeters. Then, the images with double labeling data were analyzed by Image J for statistical process or organized with Adobe Photoshop CS5 (Adobe Inc, San Jose, CA) for preparing the manuscript.

### Statistical analyses

One-way ANOVA with Newman-Keul’s post comparison for all pair of columns was used to compare CCL2-ir intensity in naïve or saline cortex, CC and CC with Ependyma (CC+Epend), and in LPS treated cortex, CC and CC+Epend. The same ANOVA and post-test were applied to compare the number of CCL2-ir and GFAP or Iba-1 or NeuN double labeled structures in the cortex, CC and CC+Epend of normal, saline and LPS injected mice. Statistics was processed with Graph Pad Prism 5 (Graph Pad Software Inc. La Jolla, CA) and the significant difference of *p* < 0.05, *p* < 0.01 and *p* < 0.001, were represented as “*”, “**” and “***”, respectively.

## Results

### CCL2-ir levels in the cortex and CC of naïve, saline and LPS injected mice

**Fig. 1:**
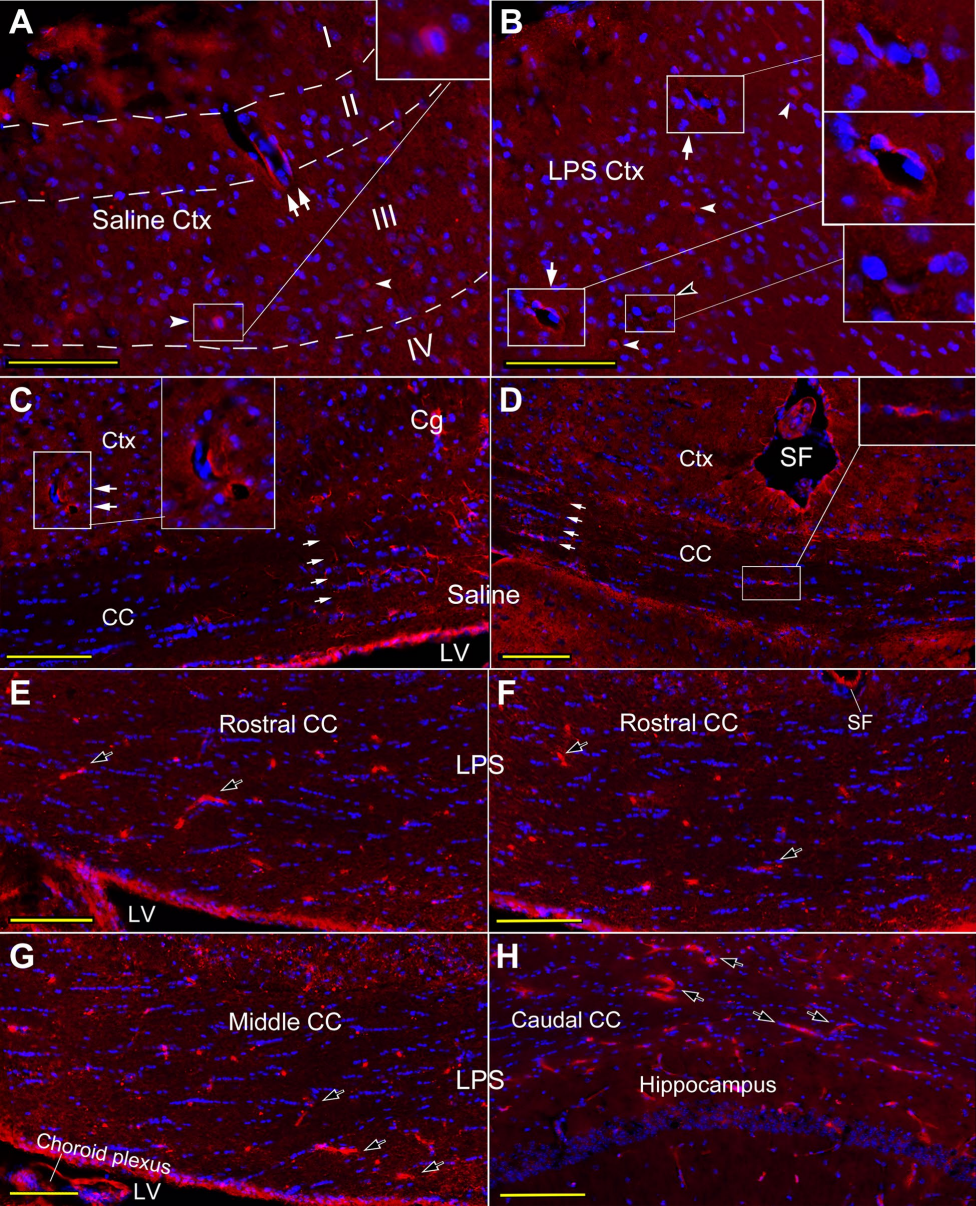
Cellular distribution of CCL2-ir in cortex and CC of saline and LPS injected mice. **A**, **B**, CCL2-ir positive structures and neuron like cells scatter in the cortex of saline (**A**) and LPS (**B**) injected mice, respectively. The former is generally situated in the area surrounding the blood vessel like profiles (arrows and insets), and the latter is mostly located in I ~ III of the cortex layers (arrowheads and insets). The vasculature with negative perivascular labeling is also observed (opened arrowhead and inset in **B**). **C**, **D**, the CC in saline injected mice, showing majority of CCL2-ir labeling are located in lateral CC between Cg and LV (aligned arrows), and these labeling are commonly surrounding or laying against vasculature like profiles, like what is seen in neighbor cortex (inset in **C**). Occasionally, the neuron-like CCL2-ir positive cell is encountered in the CC (inset in **D**). **E−H** Rostral (**E**, **F**), middle (**G**) and caudal (**H**) plane of the CC in LPS treated mice, in which a large number of CCL2-ir positive cashew shape elements were viewed scattering in entire CC WM tract in coronal planes, and the opened arrows point to the typical or larger ones. These cashew like CCL2-ir labeling seems be inside of microvasculature, and more of them are distributed in the CC (**E**– **H**, opened arrows) than in the cortex. Cg, cingulate cortex; Ctx, cortex; LV, lateral ventricle; SF, sagittal fissure. Scare bar = 100 µm in all **A**– **H**

*CCL2-ir labeling in the cortex and CC of normal or saline injected mice* The CCL2-ir labeling was overtly visualized in the immunostained sections (Fig. 1), while no visible positive structure was observed in the control stain (Additional file 2: Fig. S2A, B and E). Most of CCL2-ir positive structures were observed in perivascular region in both the cortex (Fig. 1A, B, arrows and insets) and the CC (C, D, arrows and insets). Vasculature like profile with CCL2-ir negative perivascular area was also observed from time to time (Fig. 1B, opened arrowhead and inset). Some neuron-like cells, characterized by the clear DAPI marked nuclei centered in CCL2-ir positive ovoid or fusiform contours, were encountered in the cortex (Fig. 1A, B, arrowheads and inset) or the CC (D, inset), especially in I ~ III layers of the cortex (A, dash line). In the CC, CCL2-ir labeling is predominantly situated in bilateral parts of the CC, the areas between cingulate cortex and lateral ventricles (Fig. 1C, D, aligned arrows). The staining was resulted from a combination of two non-competing CCL2 antibodies (PA5-34,505 and AB25124, see Table 1 and Additional file 1: Fig. S1).

#### CCL2-ir labeling in the cortex and CC of LPS injected mice

Distribution of CCL2-ir positive structures in the frontal cortex after systemic LPS was similar to that in normal or saline cortex (Fig. 1A and B), but it appeared that more neuron like cells co-localized with CCL2-ir labeling (B, arrowheads). Additionally, instead of been mainly situated in lateral parts of the CC, more CCL2-ir positive cashew shape elements (Fig. 1E–H, opened arrows point to typical or larger ones) were viewed spread in entire CC WM tract in coronal planes, and from rostral (E, F) to caudal (G, H) segments. These cashew shape CCL2-ir labeling were come across in the cortex either, but evidently fewer than in the CC. These cashew shape CCL2-ir positive structures exhibited microvasculature like profiles (Fig. 1E–H).

#### Statistical analysis of CCL2-ir intensities in cortex and CC of normal, saline and LPS mice

**Fig. 2:**
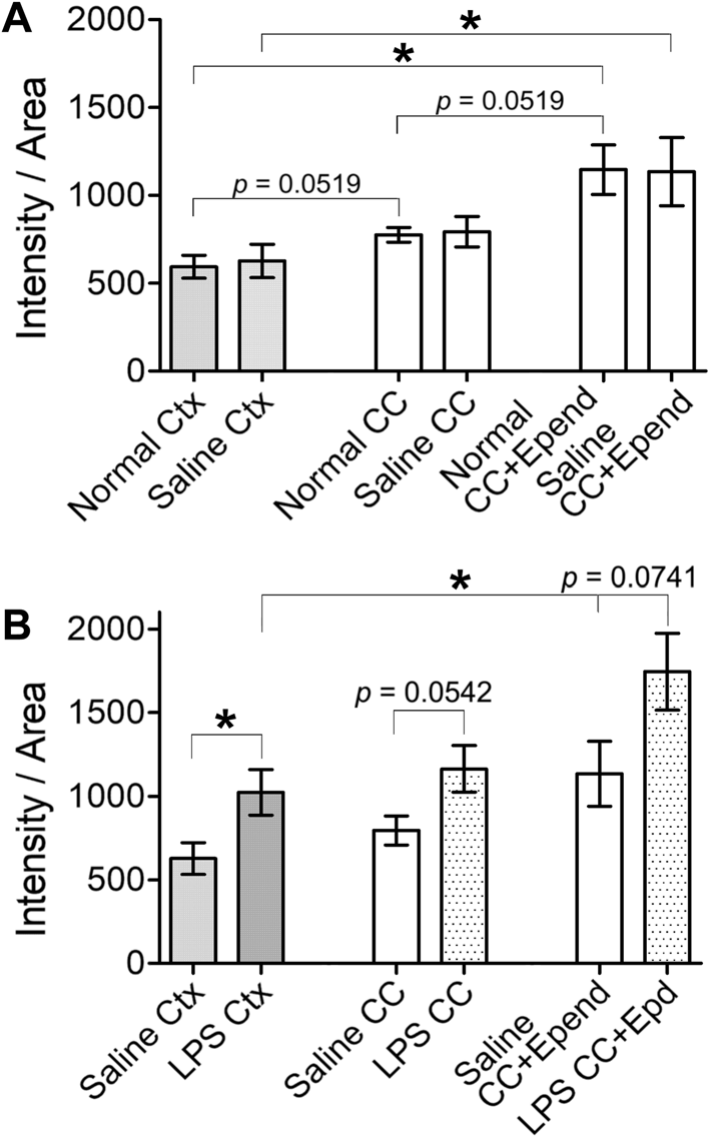
Statistical comparison of CCL2-ir intensities among cortex and CC of normal, saline and LPS injected mice. **A** ANOVA analysis shows that the intensity of CCL2-ir labeling in the CC WM is clearly higher (*p* = 0.0519) than that in the cortex GM in naïve animals; while, the intensity in the CC +Epend (of naïve/saline) is significantly higher than that in the cortex (of naïve/saline; *p* < 0.05 for both pair), but evidently higher than that in the CC (of naïve/saline) excluded ependyma (*p* = 0.0519). **B** The CCL2-ir intensity in the cortex of LPS injected mice is significantly higher than that in the saline cortex (*p* < 0.05). That intensity in the CC of LPS injected mice is evidently higher than that in the saline CC (*p* = 0.0542). Similarly, the CCL2-ir intensity in the CC+ Epend of LPS treated mice is apparently higher than that in the saline CC+ Epend (*p* = 0.0741). Only that intensity in the CC + Epend was significantly higher than in the cortex following systemic LPS (*p* < 0.05)

One-way ANOVA with Newman-Keul’s post-test resulted in that the CCL2-ir intensity in the CC WM was markedly higher than that in the cortex GM, close to a significant level (Fig. 2A , p = 0.0519). Similarly, the CCL2-ir intensity in the CC+Epend was obviously higher than that in the CC without an ependymal layer (A, *p* = 0.0519). While, the CCL2-ir intensity in the CC+Epend of both normal and saline mice were significantly higher than that in the normal and saline cortex (Fig. 2A, p < 0.05 for each). Meanwhile, CCL2-ir intensity in the cortex or CC of the normal vs saline mice showed no obvious difference (A); thus, the CCL2-ir intensity in the saline injection group can represent these data from the naïve group.

Systemic LPS significantly increased the CCL2-ir intensity in the cortex comparing to that in saline injected mice (Fig. 2B, *p* < 0.05). Whereas, the post test showed that intensity in both the CC and CC+Epend of LPS treated mice were clearly augmented but not reach to a significant level (Fig. 2B, *p* = 0.0542 in saline *vs* LPS CC; *p* = 0.0741 in saline *vs* LPS CC +Epend). Among the LPS injected mice, CCL2-ir intensity in the CC+Epend was significantly higher than that in the cortex (Fig. 2B, *p* < 0.05).

### Cellular CCL2-ir distribution in brain of normal, saline and LPS injected mice

#### Co-localization of CCL2-ir and GFAP-like labeling in the cortex and CC

**Fig. 3:**
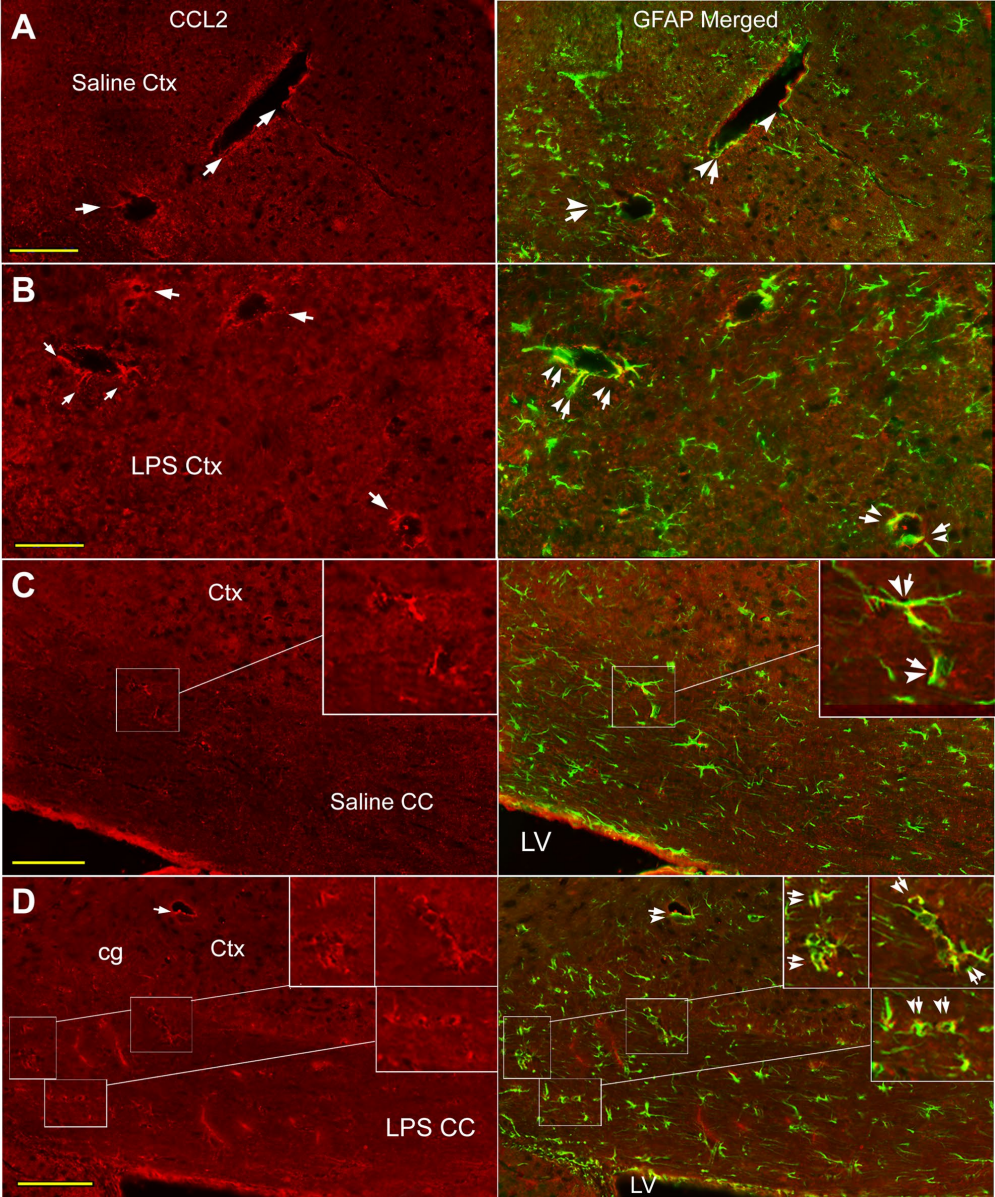
Co-localization of CCL2-ir and GFAP labeling in cortex and CC of saline and LPS injected mice. **A**, **B** In the cortex of both saline and LPS injected mice, CCL2-ir and GFAP double labeled structures (arrow-arrowheads) are mainly located in perivascular area (CCL2 panels, arrows). While, in some cases, the CCL2-ir positive structure appears to be laying against to the GFAP labeling (**A**, GFAP Merged, arrowhead). **C**, **D** Similarly, co-localizations of CCL2-ir and GFAP labeling (insets, arrow-arrowheads) are predominantly surrounding the microvasculature like structures (CCL2 panels, arrow) in the CC of both saline and LPS injected mice. Scare bar = 100 µm in all **A**– **D**

The CCL2-ir and GFAP-like double labeled structures were seen in the cortex and the CC of normal or saline injected mice (Fig. 3A, C), and these co-labeling were usually in perivascular region as circle or curve (Fig. 3A, arrows; C, framed area and inset, arrow-arrowheads). Co-localization of CCL2-ir and GFAP immunoreactivity was also observed in the ependymal layers attached to the CC (Fig. 6A, arrow-arrowhead).

Following systemic LPS, co-localization of CCL2-ir (Figs. 3B, D arrows; 5A, B arrows) and GFAP-like immunoreactivity was also mainly observed in perivascular area in both the cortex (Figs. 3B, 5A) and the CC (Figs. 3D, 5B, insets, arrow-arrowheads). However, the CCL2-ir and GFAP labeling appeared not to be always overlapped (Fig. 3A GFAP Merged, arrowhead); generally, CCL2-ir positive rings sometimes were aligned inside of GFAP labeled circles (Fig. 5A, B insets). Most of the double labeling in the CC was scattered in regions between the cingulate cortex and the lateral ventricle (Fig. 3C, D). Similarly, the CCL2-ir and GFAP double labeling was also seen abutting or inside the ependymal layer attached to the CC (Fig. 6B, arrow-arrowheads).

#### Co-localization of CCL2-ir and Iba-1-like labeling in the cortex and CC

**Fig. 4:**
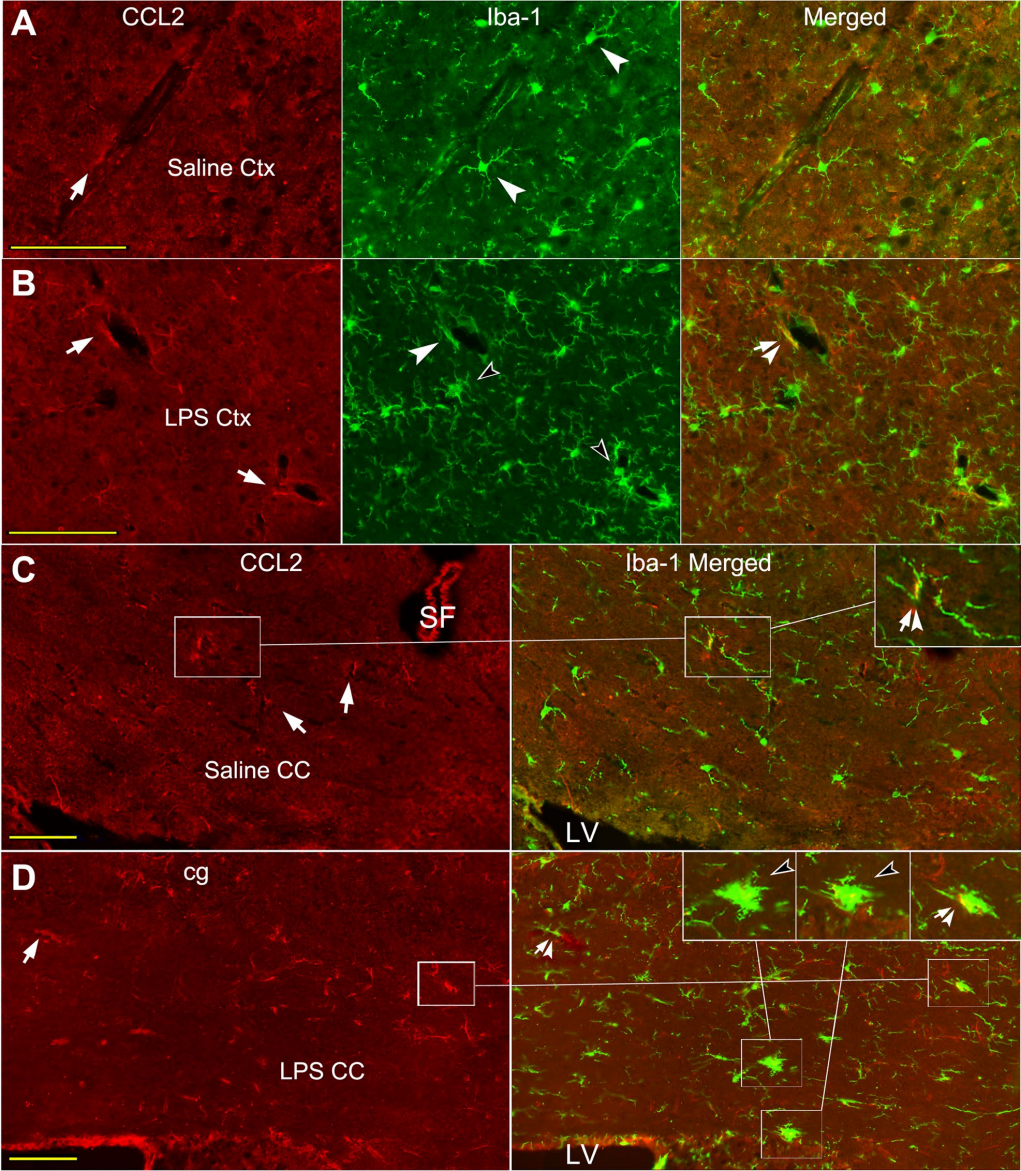
Co-localization of CCL2-ir and Iba-1 labeling in cortex and CC of saline and LPS injected mice. **A**, **B** Likewise, CCL2-ir positive structures are mainly situated in perivascular regions (CCL2 panels, arrows). Delicate ramified Iba-1 labeled cells (**A** Iba-1 panel, arrowheads) are viewed in saline injected mice; while, in LPS injected mice, hyper-ramified and/or hypertrophic soma of Iba-1 labeled cells are encountered (**B**, Iba-1 panel, opened arrowheads). The co-labeled structure (**B** Merged, arrow-arrowhead) is usually adjacent to the vasculature like profiles either, but few. **C** Co-localization of CCL2-ir and Iba-1 is occasionally visualized in the CC of saline injected mice (Iba-1 Merged, inset, arrow-arrowhead), which is also adjacent to a micro-vessel like contours (framed areas and inset). **D** In the CC of LPS injected mice, more amoeba like Iba-1 positive somata are presented (framed areas and insets, opened arrowheads), reflecting their activated states. Some of amoeba like Iba-1 labeled cells are also CCL2-ir positive (framed area and inset, arrow-arrowhead). Some pseudopodia like structures are also CCL2-ir (CCL2 panel, arrow) and Iba-1 double positive (Iba-1 Merged panel, arrow-arrowhead). Scare bar = 100 µm in all **A**–**D**

The co-localization of CCL2-ir (arrows) and Iba-1 (arrowheads) labeling was seldom visualized in the cortex and CC of naïve or saline injected animals (Fig. 4A, C); likewise, the double labeled pseudopodia like structures were occasionally seen in perivascular areas (Fig. 4C inset, arrow-arrowheads). Sporadically, CCL2-ir and Iba-1 double labeled soma (Fig. 6C, arrow-arrowhead) was seen between the CC and basal ganglion at the corner of lateral ventricle (C, framed areas and insets), which seems be adjacent to an invaginated choroid plexus tissue.

**Fig. 5:**
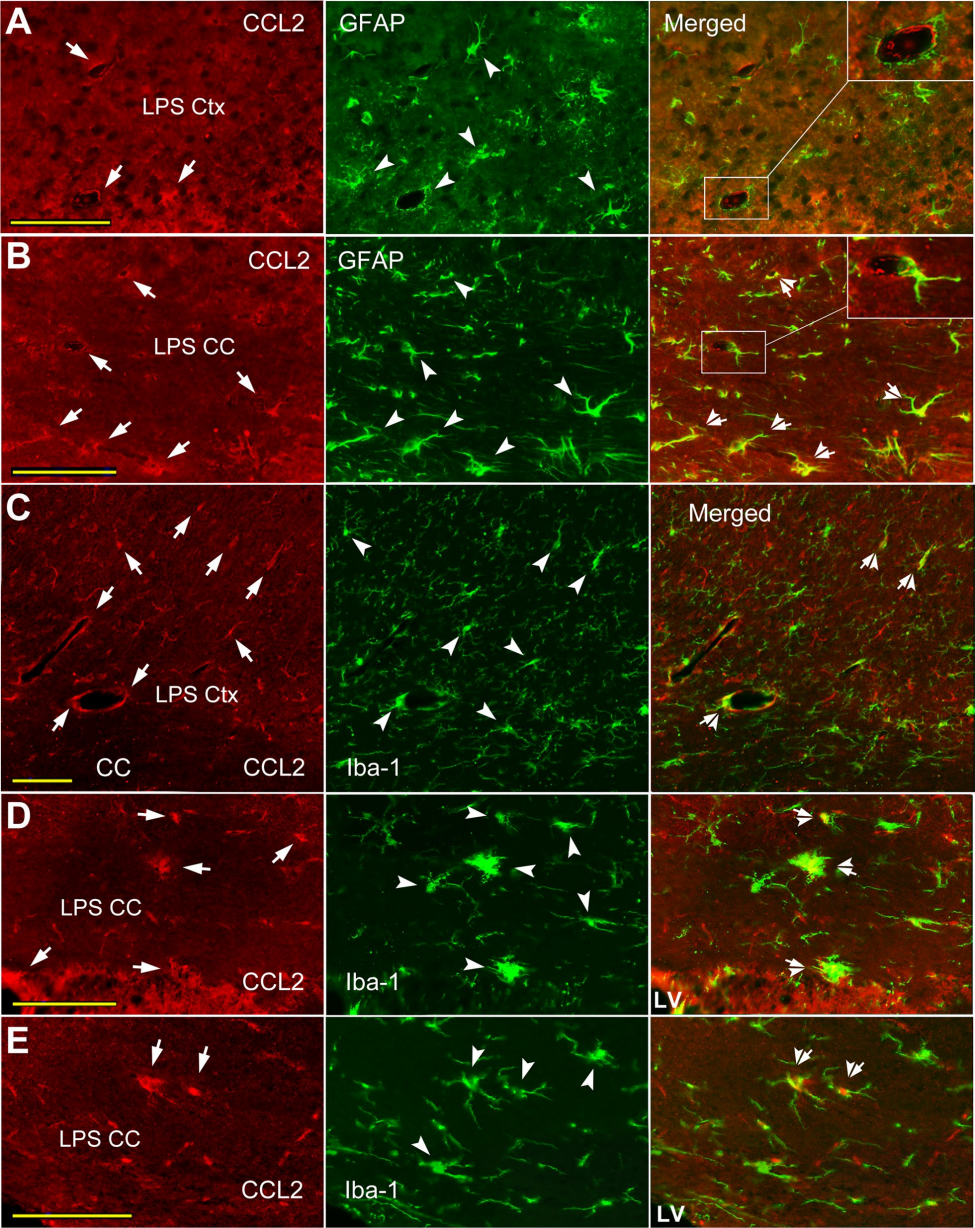
CCL2-ir and GFAP or Iba-1 co-labeled somata in cortex and CC of LPS injected mice. **A**, **B** CCL2-ir positive rings/edges or cells (CCL2 panels, arrows), and GFAP labeled rings/edges or somata (GFAP panels, arrowheads) are observed; while, a CCL2-ir positive ring is regarded to lay inside of a GFAP labeled circles (**A** Merged panel, framed area and inset). Similarly, in the CC (**B**), a CCL2-ir ring is also laying against an edge formed by an astrocyte (**B** Merged panel, framed area and inset). Some CCL2-ir and GFAP co-labeled somata or processes are viewed in the CC (arrow-arrowheads). **C**, CCL2-ir positive (arrows) Iba-1 labeled hypertrophic somata (arrowheads) are presented in the cortex after systemic LPS (arrow-arrowheads). **D**, **E** More Iba-1 labeled amoeba like somata (arrowheads) in the CC are CCL2-ir positive (arrows) following systemic LPS (arrow-arrowheads in Merged panels). Scale bar = 50 µm in all **A**–**E**

**Fig. 6:**
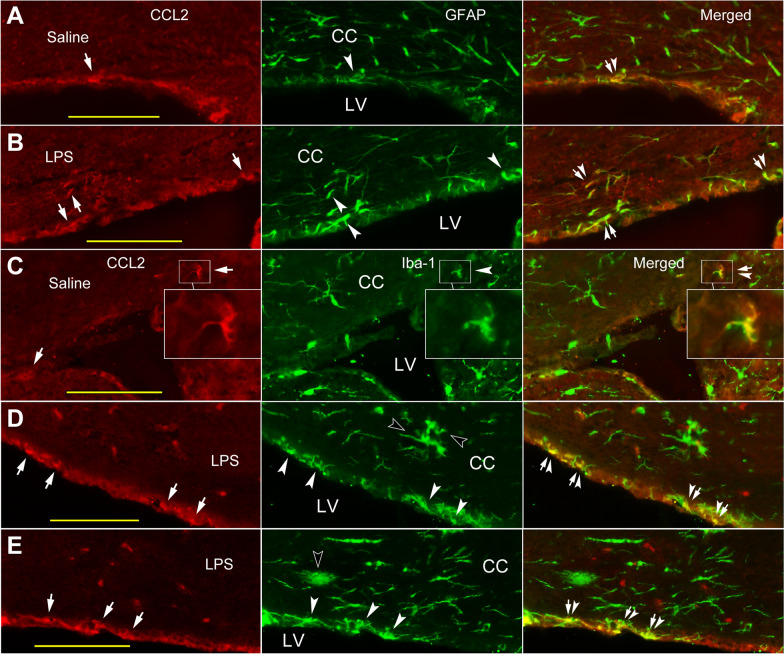
CCL2-ir cellular distribution in ependymal layers attached to the CC. **A**, **B** CCL2-ir (CCL2 panels, arrows) and GFAP (GFAP panels, arrowheads) double immunostained structures (Merged panels, arrow-arrowheads) are seen inlaid in the ependymal layers attached to the CC in both saline and LPS injected mice. **C** A CCL2-ir and Iba-1 double labeled soma (Merged, arrow-arrowhead) is seen at a corner of lateral ventricle, which seems be just next to an invaginated choroid plexus between the CC and basal ganglion (framed areas and insets). **D**, **E** Hyper-ramified and amoeba like Iba-1 labeled cells (Iba-1 panel, opened arrowheads) are observed in the CC of LPS injected mice, indicating their activated states. The CCL2-ir and Iba-1 double labeled structures are also regarded in the ependymal layers (Merged, arrow-arrowheads). Scare bar = 100 µm in all **A**– **E**

Following systemic LPS, Iba-1 positive cells with hypertrophic soma and/or hyper-ramified pseudopodia were seen in the cortex and the CC (Fig. 4B, D, opened arrowheads), and more amoeba like Iba-1 positive cells were seen in the CC (Fig. 4D, insets, opened arrowheads; Fig. 5D, E, arrowheads). This is a sign that inflammation has invaded into the brain WM, like CC. Many hyper-ramified pseudopodia (Fig. 4D, arrow-arrowhead) or amoeba like Iba-1 labeled cells were CCL2-ir co-labeled (Fig. 4D, inset, arrow-arrowheads; Fig. 5C ~ E arrow-arrowheads). The co-labeled amoeba like or hypertrophic somata were visualized in both the cortex (Fig. 5C) and the CC (D, E arrow-arrowheads), which is only occurred after systemic LPS. Meanwhile, co-localization of CCL2-ir (Fig. 6D, E, arrows) and Iba-1 (arrowheads) were identified in or adjacent to the ependymal layers (arrow-arrowheads).

#### Co-localization of CCL2-ir and NeuN-like labeling in the cortex and CC

**Fig. 7:**
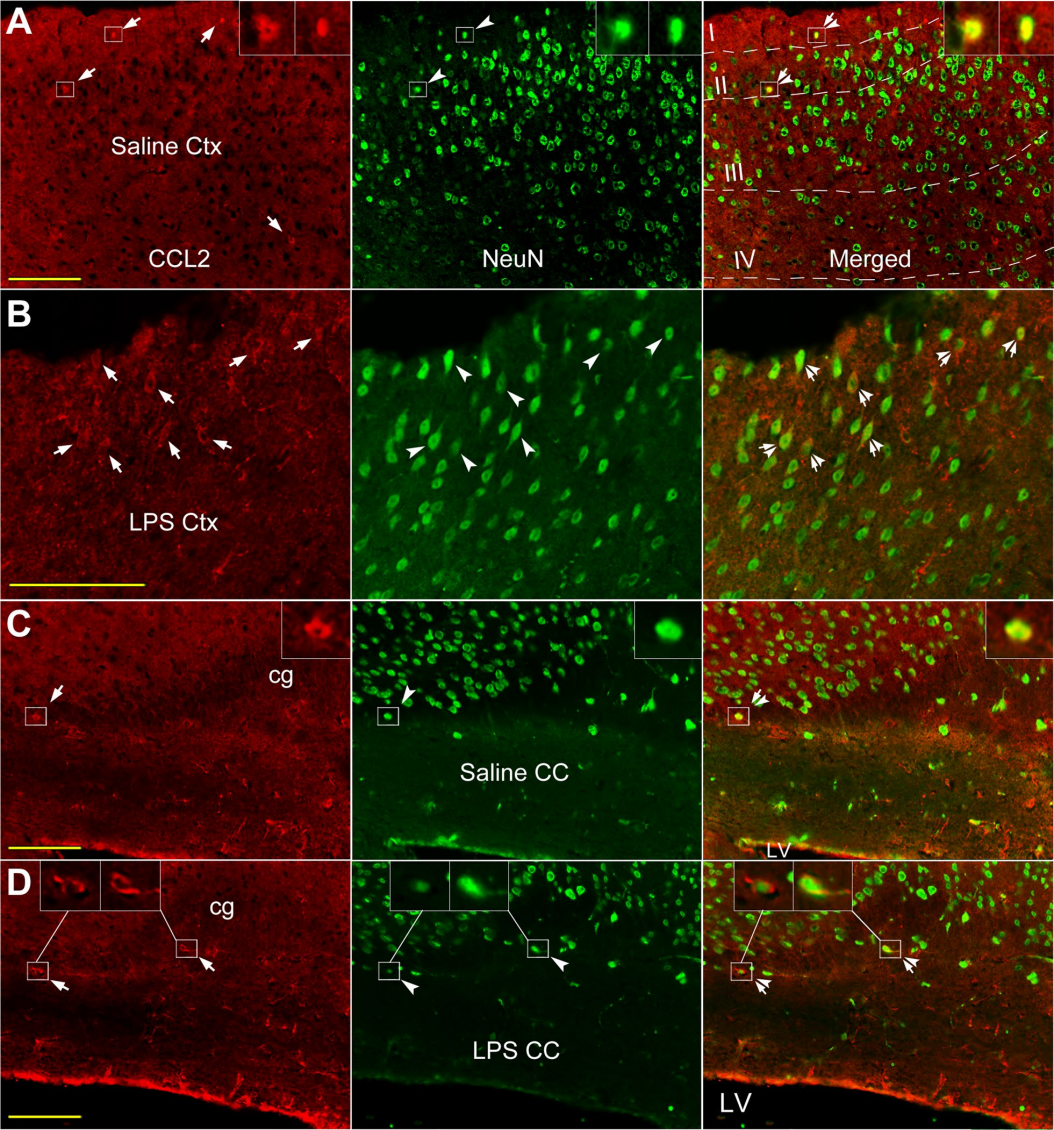
Co-localization of CCL2-ir and neuron marker in cortex and CC of saline and LPS injected mice. **A** CCL2-ir positive (CCL2 panels, arrows) neuron like (NeuN panels, arrowheads) cells (insets) are mainly situated in cortical layer I ~ III in frontal cortex (Merged panels, arrow-arrowheads, insets). **B** Following system LPS, the number of CCL2-ir and NeuN co-labeled cells (Merged, arrow-arrowheads) appears to be increased and spread broader in the cortex. **C**, **D** The CCL2-ir and NeuN co-labeled cells (arrow-arrowheads and insets) are also visualized in the CC and the cingulate cortex above the CC, in both saline and LPS injected animals. Scare bar = 100 µm in all **A**–**D**

In normal frontal cortex, most of the CCL2-ir positive (Fig. 1A, arrowheads, inset; Fig. 7A, arrows, insets) and NeuN labeled cells (Fig. 7A, arrowheads, insets) were located at I ~ III layer of the cortex (A Merged, arrow-arrowheads, insets). Normal CC seems harbor few neurons (Fig. 1D inset; Fig. 7C arrow-arrowheads, inset). Following systemic LPS, more CCL2-ir (Fig. 7B, arrows) and NeuN (arrowheads) co-labeled cells were observed in the cortex (B Merged, arrow-arrowheads). The CCL2 and NeuN double labeled cells were also seen in the CC of LPS treated mice and the above cingulate cortex (Fig. 7D, arrow-arrowheads, insets).

#### Statistical comparison of double labeling among normal, saline and LPS injected mice

**Fig. 8:**
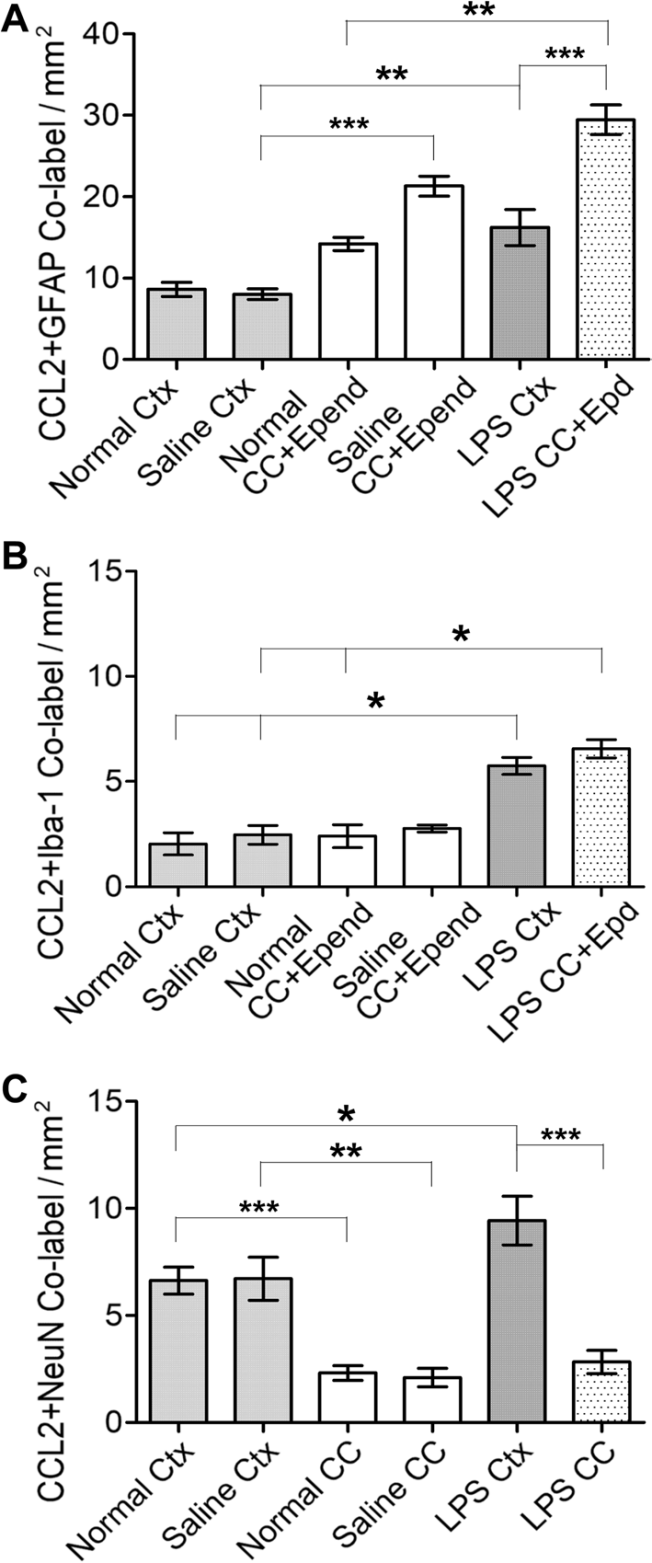
Comparison of co-localization of the CCL2-ir and the other cellular markers in cortex and CC. **A** The number of CCL2-ir and GFAP double labeled structures in saline CC+Epend is significantly higher than that in saline cortex (*p* < 0.001), revealed by ANOVA and post-test. And systemic LPS significantly enhanced the number of these co-localization in the CC+Epend comparing to its normal counterpart (*p* < 0.01). These co-localization number is also significantly increased in the cortex of LPS treated mice versus the saline cortex (*p* < 0.01). Meanwhile, in the LPS injected animals, the co-labeled structures in the CC+Epend is significantly higher than in the cortex (*p* < 0.001). **B** The number of CCL2-ir and Iba-1 co-labeled structures is significantly upgraded in both the cortex and CC+Epend of LPS treated mice, comparing to these number in both the cortex and CC +Epend of normal and saline ones (*p* < 0.05 for both). **C** The numbers of CCL2-ir and NeuN co-localization are significantly higher in naïve and saline cortex than that in naïve and saline CC (*p* < 0.001 for Ctx *vs* CC in naïve mice; *p* < 0.01 for Ctx vs CC in saline ones). Systemic LPS significantly increased these co-localizations in the cortex (*p* < 0.05) but not in the CC. Likewise, in the LPS treated mice, the number of CCL2-ir positive neurons in the cortex is significantly higher than that in the CC (*p* < 0.001)

For the CCL2+GFAP co-labeled structures, firstly, the ANOVA and post-test displayed that the number of co-localizations in the saline CC+Epend group was significantly higher than that in the saline cortex (Fig. 8, *p* < 0.001). Secondly, the systemic LPS significantly increased the number of co-localizations in both the cortex and CC+Epend (A, *p* < 0.01 in both cortex pair, and CC pair of saline *vs* LPS). Thirdly, the co-labeled structures in the CC+Epend were significantly more than that in the cortex (A, *p* < 0.001) in the systemic LPS group.

The CCL2+Iba-1 co-localizations were sporadically observed in both the cortex and CC+Epend. However, systemic LPS significantly enhanced the number of co-labeled structures in both the cortex and CC+Epend comparing with the number in the cortex and CC+Epend of the normal or saline group mice (Fig. 8B, *p* < 0.05 in both cortex pair, and CC+Epend pair of normal/saline vs LPS).

Finally, the number of CCL2+NeuN co-labeled cells in the normal or saline cortex was significantly higher than that in normal or saline CC (Fig. 8C , *p* < 0.001 for the normal pair; *p* < 0.01 for the saline pair). Then, systemic LPS significantly augmented the number of CCL2 + NeuN co-localization in the cortex (C, *p* < 0.05). Whereas, systemic LPS did not obviously change the number of these co-localizations in the CC.

## Discussion

### Different cellular CCL2-ir distributions in the frontal cortex and the CC

We had previously observed that the CCL2 peptide level in the normal rat CC and the healthy human WM was significantly higher than that in the normal GM [11]. In the present work, we also detected that the CCL2-ir intensity in the CC was distinctly higher than that intensity in the frontal cortex. Furthermore, we found that CCL2-ir labeling in ependymal layer attached to the CC contributes a substantial portion of the CCL2-ir intensities. Consequently, CCL2-ir intensity in the CC+Epend was significantly higher than that in the cortex. Several previous studies have reported either higher basic level of CCL2 mRNA in choroid plexus stromal cells [27] or measurable levels of both CCL2 mRNA and peptide in choroid plexus epithelial cells [19, 20]. Meanwhile, some previous studies implied the possibility of transmission of the CCL2 from cerebrospinal fluid (CSF) into the brain parenchyma through ependymal cells [28, 29]. It was documented that a basic level of CCL2 peptide (71 ~ 200 pg/ml) in CSF was detected either in healthy human [30, 31] or in sham treated animal cases [32]. That concentration in both the choroid plexus and CSF was dramatically increased following pathogen or mechanical stimulations [30–32]. In the current work, CCL2-ir density in the ependymal layer attached to the CC was also enhanced after systemic LPS. While, it is not clear whether the ependymal epithelial cells could actively express CCL2, or the CCL2 peptide in these cells were transferred from the CSF to the CC tissue, or vice versa. A phenomenon observed in simian immunodeficiency virus infected brain that more numerous nodular lesions (clusters of microglia or macrophage) were distributing along the ependymal layers in the CC [14] substantiates the idea that ependymal layers may harbor CCL2 peptide.

The other difference of CCL2 cellular distribution between the cortex and CC is that significantly more neurons in the cortex are CCL2-ir positive than in the CC. Further, the number of CCL2-ir and neuron marker co-localization in the cortex was significantly augmented by systemic LPS; whereas, neuron-like CCL2-ir positive cells in the CC did not obviously increase after systemic LPS. This observation might be explained by the fact that the cortex contains more neurons than the CC WM [33]. Nevertheless, the fact mentioned above implies that the CCL2 cellular origination in the GM is different from the WM in both physiological and pathological condition, though we do not know what the functional significance for this discrepancy is.

The majority of CCL2-ir positive structures in the CC are surrounding the vasculature like profiles, i.e., in perivascular area, and these CCL2-ir positive rings are predominantly situated bilaterally in lateral parts of the CC between the cingulate cortex and the lateral ventricles. The expression of CCL2 by astrocyte and/or other vascular mural cells is suggested by the fact that the CCL2-ir and GFAP labeled structures were sometimes co-localized, but sometimes layered each other surrounding vasculature like structures. Consequently, the number of CCL2-ir and GFAP double labeled structures was significantly higher in the CC+Epend than the cortex in normal animals, reflecting astrocytes and/or other vascular mural cells contribute more CCL2-ir in the CC or CC+Epend. It has been documented that, basic level of CCL2 was identified in murine and human vascular endothelial or mural cells [34–36], and vascular smooth muscle also actively expressed CCL2 once prompted by plasma cholesterol or by a few unknown soluble proteins [37, 38]. Notably, an immune-electronic microscopy study suggested layers of vascular mural cells may transmit circulating CCL2 from plasma to brain parenchyma, which forms a CCL2 density gradient from plasma to brain tissues [36]. However, which vascular mural cells actively express CCL2, and which of them underlie a transmission mechanism is still an enigma and needs further exploration.

The CCL2-ir positive astrocyte somata are occasionally encountered in both the cortex and CC, but many of them are present in the ependymal layers attached to the CC. It was documented that there exists a specific type of ependymal cells termed as tanycytes. They were originally considered to be radial glial cells inlayed in the ependymal layer but stretches their long processes far beyond the layer to play a role of molecule transferring between CSF and parenchyma or vascular system therein [28, 29]. It is possible that the CCL2-ir and GFAP double labeled cells in the ependymal layers observed in the current work are tanycytes, since tanycytes were found to be GFAP positive by a line of previous immunohistochemistry studies [29, 39]. But, it is unknown from the current work whether these tanycyte-like cells could actively synthesize the CCL2 or they just transfer the peptide between the CSF and parenchyma. Further studies are necessary to clarify these questions.

Our results showed that CCL2-ir and Iba-1 double labeled structures are few in the cortex, the CC WM and the ependymal layers of both normal and saline injected mice; occasionally, the CCL2-ir positive and Iba-1 labeled pseudopodia was visualized also in the edge of perivascular area. Reasonably, co-localization of CCL2-ir and Iba-1 labeling was significantly increased following systemic LPS, which is consistent with the previous report [40] of manifest increment of CCL2 mRNA in macrophages or microglia located in the choroid plexus, the cortex and the CC after systemic LPS. On the other hand, the major cellular components in CSF of the normal brain are T-cells and monocytes [41], and migration of Iba-1 positive cells from the CSF to the parenchyma was revealed in the other in vivo neuroinflammation model than systemic LPS [42]. This report makes us to consider the CCL2-ir and Iba-1 double labeled cells in the ependymal layers to be monocyte derived macrophages that are activated by systemic LPS.

### Distinctions of cellular CCL2-ir distribution following systemic LPS

An interesting observation in this work is that following systemic LPS, CCL2-ir profile appeared to be gathered in the small blood vessels seen as cashew like structures, which distribute more prevalently in the CC. Initially, we thought there might be some blood cells expressing CCL2 and be congested there, because neutrophils could produce CCL2 after LPS stimulation [43]. Then, we attempted to maximize the removal of the blood cells by prolonging the time of saline perfusion, but the situation did not steadily change. On the other hand, no clusters of unclear (marked by DAPI), nor accumulation of neutrophil (labeled by MPO or Ly6g) or monocyte (stained by Iba-1) were observed to co-localize with these CCL2-ir profiles within the blood vessels. These results suggest it is not the blood cells that express CCL2 and congest within the small blood vessels.

It was reported that structural feature of the vascular system in subcortical and deep WM is substantially different from that in the cortex GM; accordingly, the vasculature density and the blood volume in the GM are significantly higher than that in the WM [44–46]. Generally, arteries enter and irrigate the superficial GM at first and continue to bifurcate deeply to deep and/or subcortical WM until reaching the ependymal layers; therefore, blood flow in the WM is a kind of terminal irrigation [44–46]. It was further revealed that when the arteries travel from the cortex to the subcortical WM, like the CC, they become tapered and coiled [45]. In addition, following systemic LPS, not only plasma CCL2 elevated but also a serial of related adhesion molecules increased in the circulation, which might build up a molecular network together with the CCL2 [47, 48], especially when the vasculatures are tapering and coiling through the CC. Accordingly, we assumed that there would be some accumulation of circulating CCL2 in the terminal vasculatures after systemic LPS, which causes higher CCL2-ir intensity within these vasculatures.

The other profound impression is the change of CCL2-ir and Iba-1 co-labeled cells after systemic LPS. As aforementioned, CCL2-ir and Iba-1 co-labeled pseudopodia like structures were encountered but few in normal or saline cortex and CC, and almost none of CCL2-ir and Iba-1 co-labeled soma was identified therein. While, following systemic LPS, the CCL2-ir and Iba-1 co-localized somata were observed from time to time. Further, those double labeled somata are usually amoeba like or hypertrophic/hyper-ramified, suggesting only activated microglia or macrophages will actively synthesize the CCL2 peptide. This is consistent to the results that cultured macrophages express significantly more CCL2 peptide following LPS stimulation [49], and that in situ expression of CCL2 mRNA is upregulated in microglia after systemic LPS [40].

### Staining technique consideration

As we have observed in the present work, the majority of the CCL2-ir labeling was located in perivascular region, a kind of margin area where it is easy to precipitate none specific fluorescent protein. Therefore, we made considerable efforts to assure the CCL2 staining is valid and efficient. First of all, the specificity of the antibodies has been tested by vendors as shown in vendor’s data sheet (see Table 1 for relevant webpages), and been verified through a number of published immunostaining works, such as the staining on kidney tissue [50, 51], on liver tissue [52], on cardiac tissue [53], and on peripheral nervous tissue [54]. Technically, during immunostaining, we mounted a control section in every slide together with the other sections (Additional file 1: Fig. S1), and which was stained under exactly the same condition except without primary antibody incubation [26]. During intensity measurement, the control section in each slide was used to set up a zero-intensity background (Additional file 2: Fig. S2A, B, E) applied to all other sections stained with primary antibodies [26]. In these control staining, vasculature like structures showed clear verges (Additional file 2: Fig. S2A, B, E arrows) no matter further stained by AF 594 (A and B) or 488 (E). Yet, adding CCL2 antibody in the cocktail with either anti-Iba-1 or anti-NeuN resulted in a distinguish CCL2-ir positive perivascular circles or curves (Additional file 2: Fig.S 2C, D, F, G arrows), no matter the CCL2-ir staining was visualized by AF 594 (C, D) or by AF 488 (F, G). However, the other primary antibodies such as anti-Iba-1 or anti-NeuN did not ever precipitate in the verge area surrounding the vasculature like structures (Additional file 2: Fig. S2D’arrows; C’, F’ and G’), also, no matter they were visualized by AF 594 (C’, D’) or by AF 488 (F’, G’).

On the other hand, in a previous study on using monoclonal antibody to neutralize shedding virus equipped with host cell membrane protein, the authors demonstrated that applied two noncompeting antibodies against different epitope on the same antigenic glycoprotein would more effectively prevent immune escape of the shedding virus [55]. The authors found that this combination allows them to administer lower dose of neutralizing antibodies [55]. Accordingly, the two antibodies we had applied to stain the CCL2 are noncompeting antibodies, and they would bind to different domain of the CCL2 peptide in the N-terminal and the C-terminal (Additional file 1: Fig. S1B), separately. In order to obtain a consistent result with the same lot of antibody to finish such a large quantity of immunostaining in the current work, we tried to decrease the dose of anti-CCL2 by combining two noncompeting antibodies. Consequently, this way certainly have reduced the amount of antibody usage.

### Summarization

In summary, we indeed observed several differences in the cellular distribution of the CCL2 peptide in the frontal cortex and the CC in the current work. (1) CCL2-ir density in the CC is markedly higher than adjacent cortex, and is significantly higher when the measurement included the ependymal layer attached to the CC, which is supportive of our previous report [9] and implies that the ependymal layer contributes to a considerable quantity of CCL2-ir intensity. (2) Perivascular labeling contribute to a substantial amount of CCL2-ir intensities in both the GM and the WM, especially in the CC WM. These CCL2-ir positive circles or edges in the CC WM are distributed predominantly in lateral part of the CC between cingulate cortex and lateral ventricles. (3) After systematic LPS, more CCL2-ir positive elements appear to be accumulated within the vasculature like structures, more of them are scattered in the entire CC than in the cortex. (4) Majority of those perivascular CCL2-ir positive rings or edges are co-labeled by anti-GFAP in both the cortex and the CC, and the number of CCL2-ir and GFAP co-localization in the cortex and the CC is significantly increased following systemic LPS. (5) Few CCL2-ir and Iba-1 double labeled structure is encountered in normal cortex and CC, but the co-localization is significantly increased following systemic LPS in both the cortex and the CC. Particularly, the CCL2-ir and Iba-1 co-labeled cells are always amoeba like or with hypertrophic soma, implying only activated microglia or macrophages synthesize CCL2-ir peptide. (6) Significant more neuron-like CCL2 positive cells in the cortex contributes to CCL2-ir intensity than in the CC WM. Following systemic LPS, the number of them increased significantly in the cortex, but not in the CC. (7) The CCL2-ir + GFAP, or + Iba-1 double labeled structures are also observed inlayed in the ependymal layer attached to the CC. (8) Methodology reliability is discussed.

## Supplementary Information

**Additional file 1: Fig. S1.** Illustration of key points in method used in the present work. **A**, the control section devoid of primary antibody stain, the sections from naïve animal, and those from the saline and LPS injected animal are mounted on the same slide, in order for them to be stained in the same condition. **B**, two CCL2 antibodies have been applied together, which is PA5-34505 plus either AB25124 or NBP1-07035, because theoretically one (PA5-34505) is only binding to the C-terminals and the other (AB25124 or NBP1-07035) is predominantly binding to the N-terminals, based on the data sheet from the venders. Thus, the PA5-34505 and AB25124 or NBP1-07035 are noncompeting antibody, and this combination may decrease the amount of antibody usage during immunostaining, according to a previous study [55].

**Additional file 2: Fig. S2.** Exampling the control immunofluorescent staining. **A**, **B** and **E**, both Alex Flour 584 (**A**, **B**) or 488 (**E**) single staining are used to stain control image, in which blood vessel like profiles (arrows) are observed negative, due to without primary antibody. **C** and **C’**, the CCL2-ir labeling can be visualized surrounding some vasculature like profiles when anti-CCL2 is applied; however, the Iba-1 staining doesn’t label the perivascular areas (**C’**). **D** and **D’**, in CCL2 + NeuN double stained section, a higher intensified circle is viewed surrounding a blood vessel (aligned arrows in **D**) when anti-CCL2 is revealed by 594, but anti-NeuN plus 488 is negative in the same area (arrows in **D’**). This indicates the perivascular circle of higher intensity is not none specific binding. **F**, **F’**,** G** and **G’**, similar situation occurs when AF488 is used to mark CCL2 and AF594 to stain microglia or neurons, the same pattern of labeling is observed regardless the type of secondary antibody or fluorescent protein. Anti-CCL2 plus either 594 or 488 will result in the labeled circles or edges in perivascular regions (**F** and **G**, arrows); in contrary, neither Iba-1 nor NeuN plus any secondary antibody or relevant fluorescent protein (**F’** and **G’**) would not precipitate surrounding the vasculature like profiles.

## Data Availability

The datasets used and/or analyzed during the current study are available from the corresponding author on reasonable request.
