# Supplementary figures and images for: Cellular distribution of C-C motif chemokine ligand 2 like immunoreactivities in frontal cortex and corpus callosum of normal and lipopolysaccharide treated animal
Source: BMC Neurosci. 2022 Mar 30;23:20. doi: 10.1186/s12868-022-00706-y (PMC8965573; doi:10.1186/s12868-022-00706-y)

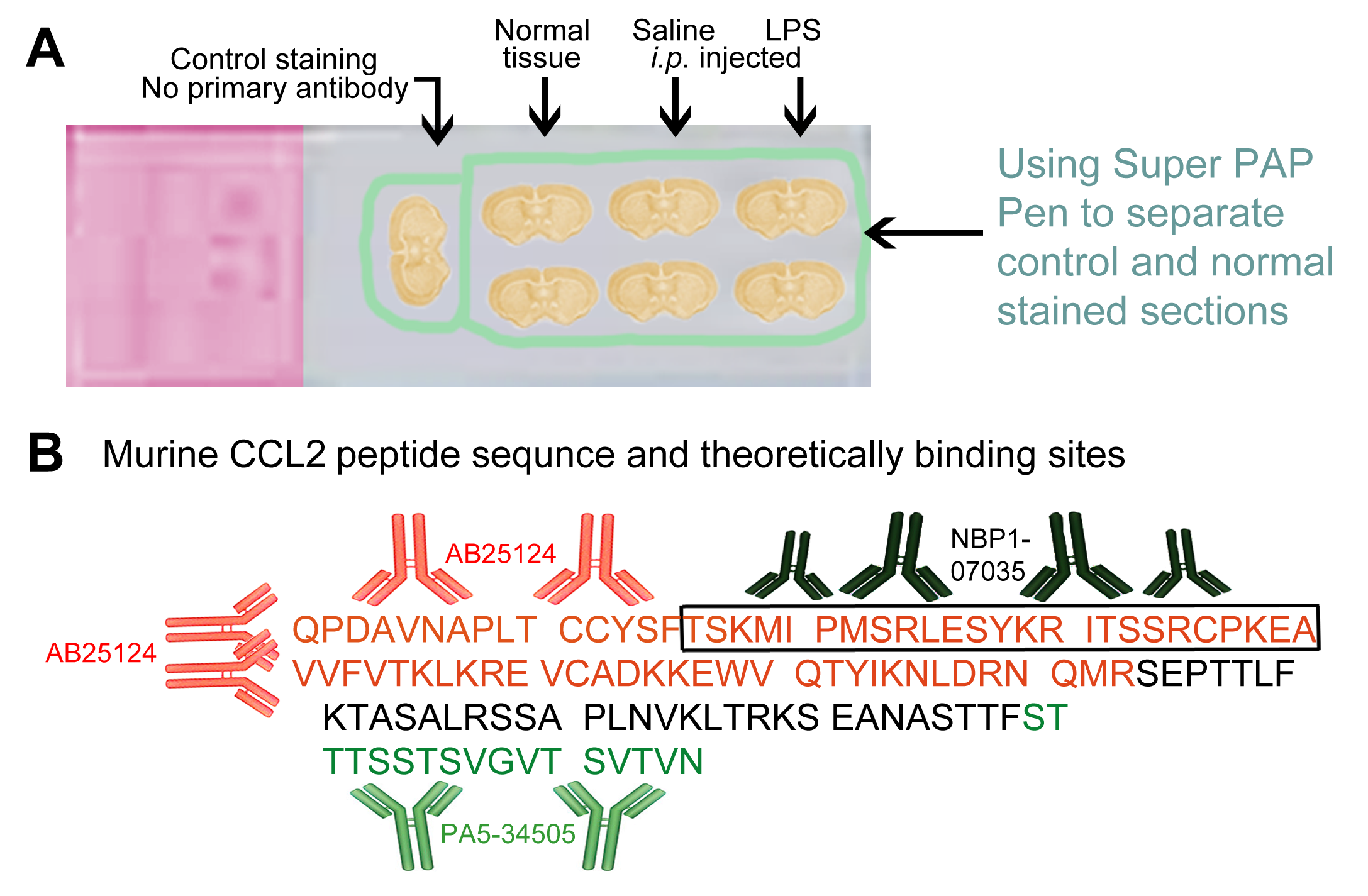

Supplement: Supplementary file 1 — Additional file 1: Fig. S1. Illustration of key points in method used in the present work. A, the control section devoid of primary antibody stain, the sections from naïve animal, and those from the saline and LPS injected animal are mounted on the same slide, in order for them to be stained in the same condition. B, two CCL2 antibodies have been applied together, which is PA5-34505 plus either AB25124 or NBP1-07035, because theoretically one (PA5-34505) is only binding to the C-terminals and the other (AB25124 or NBP1-07035) is predominantly binding to the N-terminals, based on the data sheet from the venders. Thus, the PA5-34505 and AB25124 or NBP1-07035 are noncompeting antibody, and this combination may decrease the amount of antibody usage during immunostaining, according to a previous study [55]. [file 12868_2022_706_MOESM1_ESM.tif]

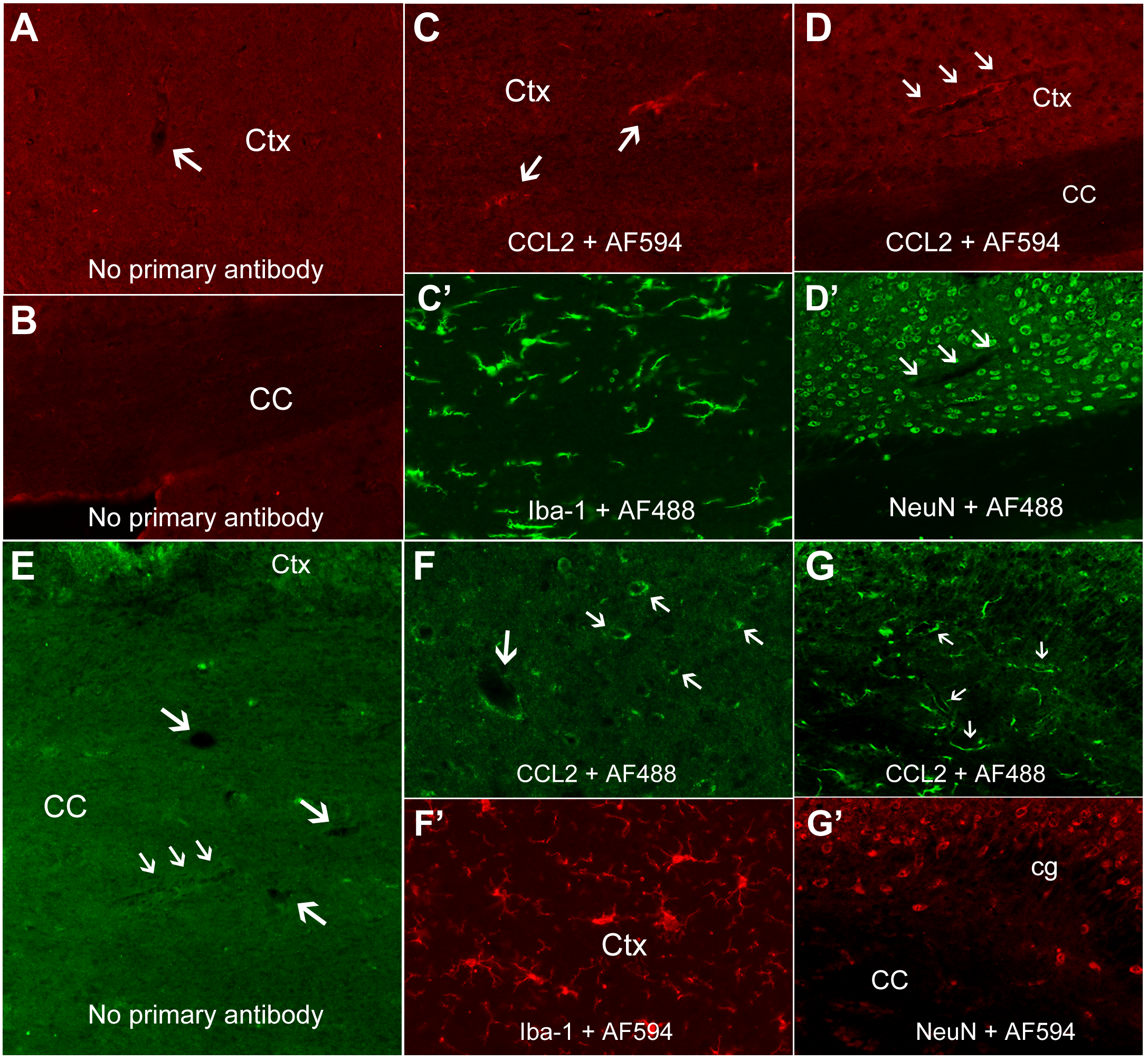

Supplement: Supplementary file 2 — Additional file 2: Fig. S2. Exampling the control immunofluorescent staining. A, B and E, both Alex Flour 584 (A, B) or 488 (E) single staining are used to stain control image, in which blood vessel like profiles (arrows) are observed negative, due to without primary antibody. C and C’, the CCL2-ir labeling can be visualized surrounding some vasculature like profiles when anti-CCL2 is applied; however, the Iba-1 staining doesn’t label the perivascular areas (C’). D and D’, in CCL2 + NeuN double stained section, a higher intensified circle is viewed surrounding a blood vessel (aligned arrows in D) when anti-CCL2 is revealed by 594, but anti-NeuN plus 488 is negative in the same area (arrows in D’). This indicates the perivascular circle of higher intensity is not none specific binding. F, F’, G and G’, similar situation occurs when AF488 is used to mark CCL2 and AF594 to stain microglia or neurons, the same pattern of labeling is observed regardless the type of secondary antibody or fluorescent protein. Anti-CCL2 plus either 594 or 488 will result in the labeled circles or edges in perivascular regions (F and G, arrows); in contrary, neither Iba-1 nor NeuN plus any secondary antibody or relevant fluorescent protein (F’ and G’) would not precipitate surrounding the vasculature like profiles. [file 12868_2022_706_MOESM2_ESM.tif]
